# Full-Length Genome Sequences of Senecavirus A from Recent Idiopathic Vesicular Disease Outbreaks in U.S. Swine

**DOI:** 10.1128/genomeA.01270-15

**Published:** 2015-11-12

**Authors:** Jianqiang Zhang, Pablo Piñeyro, Qi Chen, Ying Zheng, Ganwu Li, Christopher Rademacher, Rachel Derscheid, Baoqing Guo, Kyoung-Jin Yoon, Darin Madson, Phillip Gauger, Kent Schwartz, Karen Harmon, Daniel Linhares, Rodger Main

**Affiliations:** Department of Veterinary Diagnostic and Production Animal Medicine, College of Veterinary Medicine, Iowa State University, Ames, Iowa, USA

## Abstract

Since July 2015, vesicular lesions affecting growing pigs and sows accompanied with neonatal mortality have been reported in multiple U.S. states. Senecavirus A has been consistently detected from these cases. The complete genome sequences of 3 recent U.S. Senecavirus A isolates were determined to further characterize this virus.

## GENOME ANNOUNCEMENT

Senecavirus A (SV-A), formerly Seneca Valley virus (SVV), is a nonenveloped, single-stranded, positive-sense RNA virus belonging to the genus *Senecavirus* in the family *Picornaviridae* (1). Senecavirus A, specifically SVV-001 isolate, was initially identified as a contaminant in PER.C6 cell cultures (2). From 1988 to 2001, seven SV-A isolates were recovered from pigs in various locations in the U.S. while the clinical symptoms of these pigs were not reported in detail (3). However, there have been several refereed publications documenting identification of SV-A from pigs with idiopathic vesicular disease in Canada in 2007 (4), in the U.S. in 2010 (5), and in Brazil in 2015 (6).

Since July 2015, there have been multiple cases of vesicular diseases observed in exhibition, commercial finisher, and breeding swine herds in several U.S. states (Iowa, South Dakota, Illinois, Indiana, Missouri). Clinical presentations included vesicles on the snouts and/or coronary bands, sometimes accompanied with lameness, anorexia, lethargy, and transient fever. Affected breeding herds had an increase of neonatal (mainly piglets <7 days old) mortality ranging from 30 to 70%. Foreign animal disease investigations ruled out foot-and-mouth disease, swine vesicular disease, vesicular stomatitis, and vesicular exanthema of swine. Interestingly, SV-A was consistently detected in these cases by SV-A-specific RT-PCR. The virus was successfully isolated in ST cells (ATCC CRL-1746) from multiple cases. Three selected virus isolates (USA/IA40380/2015 from an exhibition pig in Iowa; USA/SD41901/2015 from a finisher pig in South Dakota; and USA/IA46008/2015 from a neonatal piglet in a sow farm in Iowa) were subjected to complete genome sequencing using next-generation sequencing (NGS) technology on an Illumina MiSeq platform following the procedures established in our laboratory (7). Sequences were mapped to all known picornaviruses and *de novo* assembled and then analyzed using the DNASTAR Lasergene 11 Core Suite.

The genomic sequences of USA/IA40380/2015, USA/SD41901/2015, and USA/IA46008/2015 isolates were each 7,266 nucleotides (nt) in length. The three virus isolates have similar genomic organization to previously described SV-A isolates, namely, 5′ untranslated region (UTR), a single open reading frame (ORF), and 3′ UTR. The polyprotein translated from the single ORF is predicted to be cleaved into four structural proteins (VP4, VP2, VP3, and VP1) and seven nonstructural proteins (2A, 2B, 2C, 3A, 3B, 3C, and 3D).

The complete genomes of the three SV-A isolates had 98.9 to 99.3% nt identity to each other, 93.8 to 94% to the SVV-001 isolate (GenBank accession number NC_011349), 95.9 to 96.1% to the Canadian isolate 11-55910-3 (KC667560), and 97.7 to 97.9% to the Brazilian isolates BRA/MG1/2015 and BRA/MG2/2015 (KR063107 and KR063108). The three SV-A isolates in this study had 86.9 to 93.7% nt identity to the U.S. historic SV-A isolates (EU271757 to EU271763) at the VP1 region.

The sequence data of three SV-A isolates determined in this study will facilitate future research on the epidemiology and evolutionary biology of SV-A in swine. Further study remains to be conducted to determine the association of SV-A with clinical diseases and to fulfill Koch’s postulates.

### Nucleotide sequences accession numbers.

The complete genome sequences of SV-A isolates USA/IA40380/2015, USA/SD41901/2015, and USA/IA46008/2015 have been deposited in GenBank under the accession numbers KT757280, KT757281, and KT757282, respectively.
